# Current Patterns of Management of Advanced Prostate Cancer in Routine Clinical Practice in Spain

**DOI:** 10.1155/2015/186740

**Published:** 2015-07-13

**Authors:** Maria José Ribal, Juan Ignacio Martínez-Salamanca, Camilo García Freire

**Affiliations:** ^1^Department of Urology, Hospital Clínic, Carrer Villarroel 170, 08036 Barcelona, Spain; ^2^Department of Urology, Hospital Universitario Puerta de Hierro, Calle Manuel de Falla, 1, Majadahonda, 28222 Madrid, Spain; ^3^Department of Urology, Hospital Clínico Universitario de Santiago de Compostela, Rúa da Cantaleta 9, 15706 Santiago de Compostela, Spain

## Abstract

*Objective*. To describe urologists' practice patterns when managing patients with advanced prostate cancer (PCa) in Spain. *Methods*. This was an observational study conducted by 120 urologists using retrospective data of advanced PCa patients attending hospitals and outpatient centers. *Results*. Urologists evaluated a total of 375 patients (mean age: 75 years; ECOG 0-1: 77%; mean serum PSA levels at study entry: 50.5 ng/Ml). Approximately 50% of patients had bone metastases, and 60.6% experienced pain as the main symptom of progressive disease. Primary androgen deprivation therapy (ADT) use was 99.7%, with continuous ADT as the dominant strategy (91.9%). After failure of initial ADT, antiandrogen withdrawal was the next method most commonly used in 57% of patients. Choice of secondary hormonal treatment was made mostly by urologists (96%), who continued to monitor patients. Patient follow-up after chemotherapy and supportive care were mainly done in urology units, although responsibility was shared with medical oncologists and radiologists. *Conclusion*. The urologists' attitudes towards management of PCa in the routine practice in Spain show the urologist as an integral component even when patients progress to advanced stages of the disease.

## 1. Introduction

Prostate cancer (PCa) is the second leading cause of cancer death, representing the most common malignancy in males in western countries [1, 2]. Although less than 5% of patients are in the metastatic stage at their initial diagnosis, 30–40% of patients diagnosed with localized PCa will develop metastatic disease after undergoing local therapy with curative intent [3]. The emergence of androgen deprivation therapy (ADT) constituted a significant therapeutic advance in the management of advanced PCa [4], luteinising hormone-releasing hormone (LHRH) agonists being the standard of care in ADT [5]. However, after initial good response, nearly 90% of patients will develop a castration-resistant prostate cancer (CRPC), defined as prostate cancer that progresses despite castrate levels of testosterone (<50 ng/dL; <1.7 nmol/L). At the time of CRPC diagnosis, more than 80% of patients have metastases, most commonly to bone [6]. Given the demonstrated benefit of docetaxel in metastatic CRPC patients [7], it has been and continues to be the most commonly used chemotherapeutic agent in daily clinical practice as shown in one recent survey-based study [5]. New therapeutic agents such as sipuleucel-T, abiraterone acetate, cabazitaxel, radium-223, and enzalutamide are available for metastatic CRPC prior to or after docetaxel-based chemotherapy [8–10] and have changed the management of these patients significantly [11].

As novel and potentially efficacious treatment options for patients with advanced PCa become available, a multidisciplinary management approach is crucial which necessitates shared rather than compartmentalized medical care [12]. Interaction between different specialties and with supportive physicians can establish a treatment strategy that meets each patient's needs. Urologists have traditionally played a role in the development and implementation of hormonal therapy derived from a large experience in managing PCa, which constitutes a great proportion of their practices [13]. Currently physicians in Europe seem to have a consistent management approach, with urologists being mainly involved in earlier stage PCa and oncologists in later stage disease [5]. However, there are still inconsistencies in approaches to CRPC management concerning hormonal therapy treatment patterns [5], which remains a complex decision for urologists.

The aim of this study was to understand urologists' practice patterns when managing patients with advanced PCa in Spain. This study about routine clinical practice evaluated trends and variability in treatment approaches for CRPC when managed by urologists, expanding the limited available information on common practices in PCa, which continue to be discussed and refined.

## 2. Material and Methods

### 2.1. Study Design and Patients

This was an observational study conducted by 120 urologists using retrospective data from advanced PCa patients attending hospitals and outpatient centers in Spain. Each urologist reviewed medical charts to complete a record form for all consecutive patients meeting the eligibility criteria seen during March 2012 in their usual practice. Patients older than 18 years, who had developed CRPC in the last 36 months prior to starting the study, were eligible. CRPC was defined when a patient had serum testosterone levels <50 ng/dL or <1.7 nmol/L and 3 consecutive prostate-specific antigen (PSA) rises, 1 week apart, resulting in two 50% increments over the nadir under androgen deprivation, consistent with EAU guidelines [14, 15].

All patients provided written informed consent. The study was approved by local ethics committees and conducted in accordance with the Helsinki Declaration of the World Medical Association, all its amendments, and national regulations.

### 2.2. Data Collection

The following data were collected retrospectively by participating urologists: prior treatment before progression to advanced or metastatic disease; treatment of advanced or metastatic disease including therapy for CRPC (secondary hormonal therapy, chemotherapy, and palliative treatment); specialists (urologist, radiotherapist, oncologist, or other specialists) in charge of treatment decisions for CRPC patients, their monitoring, and evaluation; number of visits conducted to the urology units and transfers between different units during patients' follow-up care. Other data included Gleason score, D'Amico risk classification, and TNM staging at diagnosis; concomitant conditions; Eastern Cooperative Oncology Group (ECOG) status (current score and at diagnosis of CRPC); PSA and testosterone levels (current and at CRPC diagnosis); sites and symptoms of metastatic cancer.

### 2.3. Statistical Analysis

There is a high real-world variability in treatment patterns among patients with prostate cancer with variations ranging from 4.0% to 49.9%, depending of the treatment modality [16]. Considering the principle of maximum variance of 50.0%, we calculated that 405 patients would need to be included in the study to estimate the proportion of treatment variation with a precision of 0.05 and an alpha risk of 0.05 in a two-sided test, allowing for a percentage of nonevaluable patients not exceeding 5%.

Data were summarized using descriptive statistics. Continuous variables were described using mean, median, standard deviation, and minimum and maximum values, and absolute frequencies and valid percentages were calculated. Statistical analyses were performed with the Statistical Package for the Social Sciences (SPSS) version 9.0 (SPSS Inc., Chicago, IL, USA).

## 3. Results

### 3.1. Patient and Disease Characteristics

A total of 120 urologists reported on 405 patients with advanced PCa. Data from 30 patients were not considered for the following reasons: no date of diagnosis of progressive disease after androgen deprivation therapy (ADT) (*n* = 12) and time between documented progression after ADT and beginning of study greater than 36 months (*n* = 18). Therefore, urologists evaluated a final population comprised of 375 patients.

Table 1 presents characteristics of CRPC patients. At first diagnosis, 186 (49.6%) patients had stage T3-T4 tumours, 105 (28.0%) had positive lymph nodes (N1), and 144 (38.4%) had distant metastases (M1). The mean ± SD time between the first diagnosis and documented metastases was 21.7 ± 30.9 months. Subsequent metastases were most likely to occur in the bones and lymph nodes in 172 (45.9%) and 89 (23.7%) patients, respectively.

### 3.2. Treatment Patterns and Clinical Management

Of the 375 patients with locally advanced or metastatic disease, 161 were treated for clinically localized prostate cancer, 68 (42.2%) with radical prostatectomy and 93 (57.8%) with radiation therapy. In patients undergoing radical prostatectomy, 19 (27.9%) patients received neoadjuvant hormonal therapy. For those receiving adjuvant therapy, hormonal therapy and radiotherapy were administered in 33 (48.5%) and 28 (41.2%) patients, respectively. In patients undergoing definitive radiotherapy, 52 (55.9%) patients received neoadjuvant hormonal treatment and 37 (39.8%) adjuvant hormonal treatment. None of these 161 patients underwent surgical castration.

For locally advanced or metastatic disease, upfront hormonal therapy was the primary approach for ADT (*n* = 374, 99.7%) (Table 2), with continuous ADT being the main strategy used in 328 (91.9%) patients for a mean of 41.5 ± 34.6 months. Intermittent androgen deprivation (IADT) was used in 29 (8.1%) patients, for a mean of 12.8 ± 9.6 months for the initial IADT period. In these cases, after a mean of treatment interruption of 11.6 ± 10.5 months, therapy was reinstituted after serum PSA levels reached a mean of 17.1 ± 34.7 ng/mL (median 4.9; interquartile range (IQR), 3.1–12.7 ng/mL). Subsequent IADT cycles had a mean duration of 11.4 ± 10.0 months.

The mean ± SD duration of ADT (intermittent and continuous) until documented progression to CRPC was 33.7 ± 34.4 months. For 346 (92.3%) cases the main indicator for defining CRPC was an increase in PSA levels with testosterone in the castration range. Radiologic progression or appearance of bone lesions and new measurable lesions (RECIST criteria 1.0) were also considered as CRPC markers in 91 (24.3%) and 56 (14.9%) cases, respectively. Once diagnosed with CRPC, 259 (70.9%) patients had an ECOG performance status of 0-1 and mean ± SD levels of PSA and testosterone of 28.9 ± 48.9 ng/mL (median 12.1; IQR, 5.2–25.2 ng/mL) and 1.3 ± 2.7 nmol/L, respectively.

After progression of the disease despite the initial ADT, urologists prescribed second hormonal therapy for 139 (38.4%) patients. Antiandrogen withdrawal was the main form of secondary hormonal manipulation used in 99 (56.9%) patients, of a total of 174 hormonal manipulations. The mean ± SD duration of secondary hormonal therapy was estimated as being 6.5 ± 6.1 months.  Table 3 summarizes the treatment modalities used after prostate cancer progression.

When managing CRPC patients, urologists were responsible for secondary hormonal manipulations in the majority of cases (167, 96.0%); oncologists (32, 18.4%) and radiation oncologists (10, 5.7%) were also involved, but to a much lesser extent. While on secondary hormonal treatment, patients were more likely to be managed by urologists; 241 (72.8%) patients were not referred to other specialists by their urologist (Table 4). Urologists performed a mean ± SD of 3.8 ± 2.1 visits every 3.1 ± 2.1 months and monitored patients using PSA measurements (*n* = 168, 96.6%), clinical examination (*n* = 133, 76.4%), and imaging tests (*n* = 92, 52.9%).

In cases of further progression after secondary hormonal therapy in CRPC patients, urologists referred half of these patients (186, 50.5%) to medical oncology for chemotherapy, of whom a majority (135, 78.9%) received first-line chemotherapy, and 22 (13.1%) needed second-line chemotherapy (Table 2). For both first-line and second-line chemotherapy, the most common regimens were docetaxel alone (*n* = 67, 49.6%;* n* = 8, 36.4%, resp.) or docetaxel in combination with prednisone (*n* = 54, 40.0%;* n* = 6, 27.3%, resp.). After completion of chemotherapy, patients were more frequently managed by urologists (*n* = 265, 83.6%) and were only referred to other physicians in 52 (16.4%) cases (Table 4).

In total, 134 (37.4%) patients needed palliative treatment, with bisphosphonates (*n* = 89, 66.4%) and analgesics being the most common agents offered. This decision was taken by urologists alone in 354 (94.4%) cases and together with oncologists (*n* = 99, 26.4%) and radiation oncologists (*n* = 35, 9.3%) in a minority of cases (Table 4). At the time of the study, patients were managed by urologists in 323 (86.1%) cases, and additionally by medical oncologists in 184 (49.1%) and radiation oncologists in 31 (8.3%) cases (Table 4).

## 4. Discussion

This study provides an overview of management practices for patients with advanced PCa in Spain as evaluated by urologists, whose role in treating advanced PCa is undergoing changes not only with novel treatment options but also with new management approaches.

Historically, the urologist has been the “hormonal therapy” specialist as involved in the initial treatment decisions for advanced or metastatic PCa [4, 17]. Here we showed that, after routine staging procedures and following the EAU guidelines [14], the majority of patients with locally advanced or metastatic disease were treated with first-line hormonal therapy, and use of continuous ADT was strongly favoured over intermittent ADT. Consequently, monitoring of adverse events that may be associated with hormonal therapy and potential biochemical progression of disease is assumed to be performed by urologists, as was the case in our study.

The traditional treatment paradigm for CRPC following initial hormonal therapy failure includes the option for secondary hormone therapy in patients with little or no evidence of metastases, followed by consideration of chemotherapy by an oncologist. In our study, when PCa progressed while being on hormonal therapy, only 38.4% of patients were considered for second hormonal treatment. This result again reflects compliance with guidelines that recommend chemotherapy in patients with extensive metastatic disease, especially those with predominant skeletal metastases [14, 15]. Remarkably, bone represented the most common site of disease progression in almost half of the study population.

The study also showed that second-line hormone decisions were mainly managed by urologists and were infrequently shared with medical and radiation oncologists. This observation could be explained by the sole inclusion of urologists in the study; however, it is to be expected given that hormonal therapies are typically managed by urologists in clinical practice [18]. The results of a recent analysis of common treatment practices in PCa across Europe showed that Spanish urologists are less involved in managing second-line hormonal therapy (39%) compared with their German (53%) or UK colleagues (80%) [5].

Current urologic practices focus on early stage disease [5]. Hence, it is not surprising that 50.8% of patients were referred to a medical oncologist for chemotherapy when CRPC progressed on hormonal therapy and that 78.9% of the referred subjects received it, mainly based on docetaxel, the standard first-line option for these cases. Nevertheless, the fact that most of these patients were subsequently managed by urologists reflects the close monitoring of patients in their advanced disease stages in routine urological practice, probably due to the vast experience and knowledge of the patient's situation from initial diagnosis [19]. As part of a comprehensive clinical management approach at this stage, which also requires many interventional and supportive therapies, other specialists were involved and comprised medical oncologists, radiologists, neurosurgeons, psychiatrists, and pain and palliative care experts.

One point of note in this study is that only just over one-third of CRPC patients with extensive metastases and painful bone metastases received palliative management, despite recommendations in the literature that the prevention or reduction of bone metastases complications is an important treatment goal to improve patient's quality of life and functional independence [20]. Although the timing of initiating pharmacotherapy for bone complications remains at the physician's discretion, EAU guidelines at the time of the study recommended the early use of bisphosphonates to prevent skeletal events and early palliative radiotherapy or analgesics for reducing pain [15]. The number of patients who were undertreated (>50%) in our study might be in part explained by the physician uncertainties about the indication, clinical benefit, and toxicity of these agents that still persist in current clinical practice, particularly regarding the use of bisphosphonates to prevent complications related to bone disease [21]. In fact, a panel of European experts agrees that not every patient with CRPC and bone metastases should be treated with a bone-modifying agent [22]. Among treated patients in our study we found high use of bisphosphonates (66.4%) compared with the overall rate obtained in a European physician survey (31.4%) [23], although the rate was similar to that specifically reported for Spain [23] and that reported in an equivalent US study (49%) [24].

Urologists are aware of the difficulty of selecting not only the right therapy, but also the right time to administer that therapy to the right patient, which leads them to carefully evaluate their patients making an individual balance of potential benefits and risk to get the best possible outcome [21]. In this study, urologists took palliative treatment decisions for almost 95% of cases, more frequently than medical oncologists (26.4%) and radiotherapists (9.3%).

It is clear that integrated patient management via a multidisciplinary approach is essential in urooncologic practice. With an in-depth understanding of the clinical course and management of CRPC and patient's history, the coordinated care provided by urologists and physicians routinely treating CRPC patients and other relevant professionals is essential to formulate an accurate strategic management plan and provide valuable and consistent advice to the patient [25]. This concept is reinforced as evidence of benefits from early use of chemotherapy increases [26, 27] and newer therapeutics options become more widely adopted [12].

It is predicted that in addition to the efficacy of novel therapies on delaying disease progression, improving quality of life and increasing overall survival [28], their optimal use in sequence or combination will offer further improvements for patients with CRPC [29]. So a change in the trends of advanced prostate cancer is proposed [30], with more engagement of urologists to manage emerging therapies and establish novel approaches to hormonal manipulation. This will require urologists to be knowledgeable about the rationale for when and how to use these newer agents and the practical aspects for their application in urology.

Apart from limitations inherent to all observational studies, the major limitation in our study is that it was conducted entirely by urologists, and consequently data are dependent on their clinical practice; thus, we cannot extrapolate our findings to other specialities routinely involved in CRPC management, and conclusions identifying urologists as having primary responsibility for managing CRPC may be positively biased. Furthermore, the study was carried out in 2012, and subsequently clinical practice may have evolved since our results were collected and analyzed.

## 5. Conclusions

This study, conducted in routine clinical practice, describes management approaches and characteristics of PCa patients representative of the population seen in urology units in Spain. It is evident that the role of urologists in managing patients with PCa in Spain today extends beyond its traditional place in early stage disease. As well as this being what prepares them for the management beyond the initial therapy it also qualifies them for dealing with decisions concerning complex situations in partnership with other clinicians.

In addition to showing the urologist as an integral component of patient management, these results reflect the changing attitudes of urologists towards managing PCa patients, which mirror the emerging treatment approaches for PCa. At a time when the models or urology practices are changing, these challenges facing urologists will have a profound role in coordinating care as well as in providing support to the patients, above all because most of them will return to their urologists for advice on therapies.

From our point of view as clinicians and given the current international trend toward the use of guidelines for ensuring that cancer management is multidisciplinary, the practice in Spain should not greatly vary across countries, at least between those without too many differences in their health systems.

## Figures and Tables

**Table 1:** Patient characteristics and disease status at diagnosis and at data analysis.

Characteristics	*N* = 375
Age, years; mean ± SD	74.8 ± 7.1
Comorbidities; *n* (%)^†^	230 (61.7)
ECOG performance status, 0-1; *n* (%)^†^	284 (76.9)
PSA, ng/mL; mean ± SD	50.5 ± 135.6
Gleason score at diagnosis; *n* (%)^†^	
2–6	50 (13.8)
7	140 (38.6)
8–10	173 (47.7)
D'Amico risk classification at diagnosis; *n* (%)^†^	
Low	23 (6.4)
Intermediate	97 (27.2)
High	237 (66.4)
Extent of disease; *n* (%)	
Bone metastases	172 (45.9)
Lymph node	89 (23.7)
Lung metastases	22 (5.9)
Pain associated with disseminated disease; *n* (%)	227 (60.6)

^†^Missing data on variable: comorbidities, *n* = 2; ECOG, *n* = 6; Gleason score, *n* = 12; D'Amico risk group classification, *n* = 18. SD: standard deviation; PCa: prostate cancer; ECOG: Eastern Cooperative Oncology Group; PSA: prostate specific antigen.

**Table 2:** Treatment decisions for management of advanced prostate cancer.

	Value
Initial treatment for advanced or metastatic disease; *n* (%)^†^	*n* = 375

Primary hormonal therapy	374 (99.7)
LHRH analogues	372 (99.5)
Nonsteroidal antiandrogens	306 (81.8)
Steroidal antiandrogens	12 (3.2)
Orchiectomy	5 (1.3)

Treatment for castration-resistant prostate cancer; *n* (%)^†^	*n* = 375

Secondary hormonal therapy	139 (38.4)
First-line chemotherapy^‡^	135 (78.9)
Second-line chemotherapy^‡^	22 (13.1)
Palliative treatment	134 (37.4)

^†^Patients may have received more than one therapeutic option. ^‡^Percentages on the number of patients referred to the oncology unit for chemotherapy (*n* = 186). Missing data for the following variables: secondary hormonal therapy, *n* = 13; first-line chemotherapy, *n* = 15; second line chemotherapy, *n* = 18; palliative treatment, *n* = 17. LHRH: luteinising hormone-releasing hormone.

**Table 3:** Secondary hormonal treatment approaches (*n* = 174).

	Value
Antiandrogen withdrawal; *n* (%)	99 (56.9)

LHRH agonists; *n* (%)	59 (33.9)
Continue the patient on initial LHRH agonist	51 (86.4)
Switch to a different LHRH agonist	8 (13.6)

Addition of antiandrogens; *n* (%)	47 (27.0)
Nonsteroidal antiandrogens	41 (87.2)
Steroidal antiandrogens	6 (12.8)

Adrenal testosterone inhibitors; *n* (%)^†^	26 (14.9)
Ketoconazole	21 (84.0)
Corticoids	4 (16.0)

Continue the patient on initial treatment; *n* (%)	25 (14.4)

Antiandrogen replacement; *n* (%)	12 (6.9)
Nonsteroids	8 (66.7)
Steroids	4 (33.3)

Estrogenic compounds	8 (4.6)

Data are expressed as *n* (percentage of total second hormonal manipulation and option used). Percentages may add up more than 100% as patients could receive more than one hormonal manipulation. LHRH: luteinising hormone-releasing hormone. ^†^One missing data.

**Table 4:** Specialists involved in the therapeutic decision-making process^†^.

	Urology Unit	Radiation and Medical Oncology Unit/other services^‡^
Hormonal treatment manipulations decisions; *n* (%)	167 (96.0)	42 (24.1)
Patient follow-up after secondary hormonal treatment; *n* (%)^§^	241 (72.8)	90 (27.2)
Patient follow-up after chemotherapy; *n* (%)^§^	265 (83.6)	52 (16.4)
Palliative treatment decisions; *n* (%)	354 (94.4)	134 (35.7)
Patient follow-up at the time of the study; *n* (%)	323 (86.1)	215 (57.4)

^†^Patients may have been seen by more than one specialist. Valid percentages are presented. ^‡^Other services include the Pain and Palliative Care Units, Psychiatry, and Neurosurgery. ^§^Percentage of the total population. Variables with missing data: patient follow-up after secondary hormonal treatment, *n* = 44; patient follow-up after chemotherapy, *n* = 58.

## References

[1] Amaral T. M., Macedo D., Fernandes I., Costa L. (2012). Castration-resistant prostate cancer: mechanisms, targets, and treatment. *Prostate Cancer*.

[2] Jemal A., Siegel R., Xu J., Ward E. (2010). Cancer statistics, 2010. *CA Cancer Journal for Clinicians*.

[3] Shapiro D., Tareen B. (2012). Current and emerging treatments in the management of castration-resistant prostate cancer. *Expert Review of Anticancer Therapy*.

[4] Molina A., Belldegrun A. (2011). Novel therapeutic strategies for castration resistant prostate cancer: inhibition of persistent androgen production and androgen receptor mediated signaling. *Journal of Urology*.

[5] Sternberg C. N., Baskin-Bey E. S., Watson M., Worsfold A., Rider A., Tombal B. (2013). Treatment patterns and characteristics of European patients with castration-resistant prostate cancer. *BMC Urology*.

[6] Kirby M., Hirst C., Crawford E. D. (2011). Characterising the castration-resistant prostate cancer population: a systematic review. *International Journal of Clinical Practice*.

[7] Tannock I. F., de Wit R., Berry W. R. (2004). Docetaxel plus prednisone or mitoxantrone plus prednisone for advanced prostate cancer. *The New England Journal of Medicine*.

[8] European Association of Urology Guidelines on Prostate Cancer 2013. http://uroweb.org/wp-content/uploads/09_Prostate_Cancer_LR.pdf.

[9] Heidenreich A., Bastian P. J., Bellmunt J. (2014). EAU guidelines on prostate cancer. Part II: treatment of advanced, relapsing, and castration-resistant prostate cancer. *European Urology*.

[10] Mohler J. L., Kantoff P. W., Armstrong A. J. (2013). Prostate cancer, version 1.2014. *Journal of the National Comprehensive Cancer Network*.

[11] Heidenreich A., Porres D., Piper C., Thissen A. K., Pfister D. (2013). Metastatic castration-resistant prostate cancer: integrating new learnings to optimise treatment outcomes. *Minerva Urologica e Nefrologica*.

[12] Shore N. D. (2013). Chemotherapy for prostate cancer: when should a urologist refer a patient to a medical oncologist?. *Prostate Cancer and Prostatic Diseases*.

[13] Crawford E. D. (2003). The role of the urologist in treating patients with hormone-refractory prostate cancer. *Reviews in Urology*.

[14] Mottet N., Bellmunt J., Bolla M. (2011). EAU guidelines on prostate cancer. Part II: treatment of advanced, relapsing, and castration-resistant prostate cancer. *European Urology*.

[15] European Association of Urology http://uroweb.org/wp-content/uploads/08_Prostate_Cancer-September-22nd-2011.pdf.

[16] Cooperberg M. R., Broering J. M., Carroll P. R. (2010). Time trends and local variation in primary treatment of localized prostate cancer. *Journal of Clinical Oncology*.

[17] Sharifi N., Gulley J. L., Dahut W. L. (2005). Androgen deprivation therapy for prostate cancer. *Journal of the American Medical Association*.

[18] Villavicencio H., Hernández C., Gómez A. (2013). Treatment of prostate and renal cancer with oral drugs (abiratarone and antiangiogenic agents): positioning statement from the Spanish Association of Urology. *Actas Urologicas Espanolas*.

[19] Taneja S. S. (2003). A multidisciplinary approach to the management of hormone-refractory prostate cancer. *Reviews in Urology*.

[20] Carter J. A., Botteman M. F. (2012). Health-economic review of zoledronic acid for the management of skeletal-related events in bone-metastatic prostate cancer. *Expert Review of Pharmacoeconomics and Outcomes Research*.

[21] Clarke N. W. (2014). Balancing toxicity and efficacy: learning from trials and treatment using antiresorptive therapy in prostate cancer. *European Urology*.

[22] Fitzpatrick J. M., Bellmunt J., Fizazi K. (2014). Optimal management of metastatic castration-resistant prostate cancer: highlights from a European Expert Consensus Panel. *European Journal of Cancer*.

[23] Pokras S., Zyczynski T., Lees M., Jiao X., Blanchette C., Powers J. (2013). Treatment patterns after castration resistant prostate cancer (CRPC) diagnosis: a European physician survey. *Value in Health*.

[24] Freedland S. J., Richhariya A., Wang H., Chung K., Shore N. D. (2012). Treatment patterns in patients with prostate cancer and bone metastasis among us community-based urology group practices. *Urology*.

[25] Sternberg C. N., Krainer M., Oh W. K. (2007). The medical management of prostate cancer: a multidisciplinary team approach. *BJU International*.

[26] Armstrong A. J., George D. J. (2010). Optimizing the use of docetaxel in men with castration-resistant metastatic prostate cancer. *Prostate Cancer and Prostatic Diseases*.

[27] Berthold D. R., Pond G. R., Soban F., de Wit R., Eisenberger M., Tannock I. F. (2008). Docetaxel plus prednisone or mitoxantrone plus prednisone for advanced prostate cancer: updated survival in the TAX 327 study. *Journal of Clinical Oncology*.

[28] Thoreson G. R., Gayed B. A., Chung P. H., Raj G. V. (2014). Emerging therapies in castration resistant prostate cancer. *The Canadian Journal of Urology*.

[29] Logothetis C. J. (2013). Treatment of castrate-resistant prostate cancer. *Journal of Urology*.

[30] Woo H. H. (2013). Prostate cancer: are urologists ready to manage castration-resistant disease?. *Nature Reviews Urology*.

